# We Need More Continuity Training

**DOI:** 10.7759/cureus.24106

**Published:** 2022-04-13

**Authors:** Benjamin R Doolittle

**Affiliations:** 1 Internal Medicine-Pediatrics, Yale University School of Medicine, New Haven, USA

**Keywords:** acgme core competencies, primary medical care, medical resident education, continuity clinic, ambulatory training

## Abstract

We need to increase the continuity ambulatory component in medicine, pediatrics, and med-peds program requirements. I believe our curriculum has tilted too far towards inpatient training. We are grooming a generation of hospitalists and hospital-based practitioners at the expense of our outpatient training. Increasing continuity ambulatory training offers an important opportunity for autonomy, direct patient care and vocational formation.

## Editorial

We need to increase the continuity ambulatory component in medicine, pediatrics, and med-peds program requirements. I believe that our curriculum has tilted too far towards inpatient training. We are grooming a generation of hospitalists and hospital-based practitioners at the expense of our outpatient training. For internal medicine, the Accreditation Council for Graduate Medical Education (ACGME) requires 130 half-day clinic sessions over 30 months of training, or only 65 total days during training [1]. For pediatrics and medicine-pediatrics programs, the requirement is 36 half-day clinics over 26 weeks, only 18 days over half a year [2,3]. We certainly could not teach our residents inpatient medicine with so few days. It is short-sighted and naïve to believe we can do the same in the ambulatory setting. 

Ambulatory continuity clinic is an important opportunity for trainee autonomy, direct patient care, and vocational formation. The continuity practice is an important moment when a trainee is alone with a patient for a sustained period. In the inpatient setting, trainees are almost always in the presence of a resident colleague, an attending, or a nurse. This can be helpful for on-the-fly teaching and modeling of behaviors, but it does not allow the same one-on-one connection with a patient. While one-third of medicine training is designated as ambulatory, much of those experiences are hospital-based, such as in the emergency department, which detracts from the sense of ownership found in continuity practice. 

In the ambulatory setting, there is a higher ratio of patient contact than the inpatient setting. In an afternoon session, a resident will spend several hours directly with patients. In the inpatient setting, after rounds, residents spend much of their time in front of a computer or communicating with specialists, discharge planners, nurses, and the like. One study showed that interns on inpatient medicine rotations engaged in direct patient care only 13% of the time [4]. While there are no relevant time-studies for ambulatory practice, my own sense is that residents spend far more time directly with patients. The clinic is a crucial space for a trainee to learn how to be a doctor. 

I think a reasonable goal would be to increase the continuity clinic component to 30% of residency training. This would require some give on the inpatient side as well as other ambulatory experiences, but still allow for the breadth and depth of important clinical experiences. The continuity experience needs to feel like a home-base for trainees, a place of continuity, autonomy, and support. X+Y schedules show promise for this, but often the non-inpatient component schedules didactic sessions and other activities rather than ambulatory clinics, which remain a small percentage of overall training. Continuity training cannot be the afterthought of ward rotations. My own belief is that increased continuity clinic exposure would increase ownership of the clinical experience, deepen patient-physician connection, and improve resident well-being through higher-quality experiences. 

Increasing continuity clinic is not only good for trainees, it is good for patients. Twenty-five percent of Americans do not have a primary care physician, a number that has been increasing [5]. An increased ambulatory training footprint among trainees would fulfill a national need. Further, improving the skill of residents with continuity clinic could potentially increase the numbers going into primary care. In one study, improving the continuity clinic experiences was associated with the increased likelihood of residents entering primary care [6]. I fear that inpatient service needs are compromising our residents’ education and the health of our country. Increasing the component of quality ambulatory continuity experiences responds to this need by fostering autonomous trainee growth and high-quality direct patient care. 
